# *Dolichos kongkandae* sp. nov. and lectotypification of *D. fragrans* (Leguminosae, Papilionoideae) from Asia

**DOI:** 10.3897/phytokeys.175.57759

**Published:** 2021-03-19

**Authors:** Rumrada Meeboonya, Chatchai Ngernsaengsaruay, Henrik Balslev

**Affiliations:** 1 Department of Botany, Faculty of Science, Kasetsart University, Chatuchak, Bangkok 10900, Thailand Kasetsart University Bangkok Thailand; 2 Ecoinformatics and Biodiversity, Bioscience, Aarhus University, Build. 1540, Ny Munkegade 116, DK-8000 Aarhus C., Denmark Aarhus University Aarhus Denmark

**Keywords:** Fabaceae, lectotype, new species discovery, Phaseoleae, Phaseolinae, taxonomy

## Abstract

*Dolichos kongkandae* is described as a new species from Asia and includes a line drawing, photographs and information on its distribution and ecology. The morphological differences between *D. kongkandae* and the morphologically similar *D. tenuicaulis* are highlighted and clarified. Additionally, a lectotype for *D. fragrans* is designated.

## Introduction

*Dolichos* L. is a large genus in subtribe Phaseolinae, tribe Phaseoleae, subfamily Papilionoideae in Leguminosae (Lackey 1981; Schrire 2005). Its approximately 55 species mostly occur in Africa and only 5–6 species are found in Asia, including the Indian subcontinent, South China, Indo-China and Malesia (Schrire 2005).

There are several important diagnostic features of *Dolichos*. These include basifixed stipules, trifoliolate pinnately compound leaves, purple, purplish-pink or white flowers, a standard petal with short cone-shaped appendages and inflexed auricles, incurved, but not twisted keels, a terete and incurved upward-bending style with a short pubescent or bearded inner side of the apex, a terminal and capitate stigma, compressed fruits and reticulate pollen (Verdcourt 1970, 1978, 1979; Lackey 1981; Moteetee and Van Wyk 2012).

Four species of *Dolichos* were reported for Thailand, including *D. lablab* L., *D. schomburgkii* Gagnep., *D. subcarnosus* Wight & Arn. and *D. tenuicaulis* (Baker) Craib (Craib 1928). Of these, two species have been transferred to other genera: *D. lablab* is now a synonym of *Lablab purpureus* (L.) Sweet and *D. schomburgkii* is now a synonym of *Dysolobium dolichoides* (Roxb.) Prain.

During fieldwork at Doi Chiang Dao Wildlife Sanctuary in 2013 and 2017, a population of *Dolichos* that presented distinct dark purple flowers was discovered along the Khun Huai Mae Kok Ranger Station (Den Ya Khat) nature trail. We studied and compared this plant with available herbarium specimens of *Dolichos* and found that it was similar to specimens identified as *D. tenuicaulis*. After thorough examination of the type specimens and first publication of *D. tenuicaulis*, we found that our newly-collected plant and several of the herbarium specimens identified as *D. tenuicaulis* were not similar to the type specimens of *D. tenuicaulis* and that they differed from all the known species of *Dolichos*.

Here, we describe these collections as a new species, *Dolichos kongkandae* R. Meeboonya, Ngerns. & Balslev, with descriptions of its morphological features and we provide a line drawing and fields photographs. As part of our investigation, we found that *D. fragrans* Kerr, an endemic species from the Doi Chiang Dao Wildlife Sanctuary of Thailand, needed a lectotypification, which is consequently provided here.

### Materials and methods

The morphological observations and description of this new species is based on field collections and herbarium specimens deposited at AAU, BK, BM, BKF, CMUB, E, K, L and P which were used to compare the new species to already-known species of *Dolichos*, especially the morphologically similar species and we provide the species’ geographical information. Herbarium acronyms follow Index Herbariorum (Thiers 2019). Regarding the lectotypification of *Dolichos fragrans*, we designated a lectotype from five syntypes found at BM, E, K, L and P.

## Taxonomic treatment

### New species

#### Dolichos kongkandae

Taxon classificationPlantaeFabalesFabaceae

Meeboonya, Ngerns. & Balslev sp. nov.

927303B8-7122-5645-A0AC-ED63A61CAEA8

urn:lsid:ipni.org:names:77215900-1

Figs 1 , 2

##### Diagnosis.

*Dolichos kongkandae* is most similar to *D. tenuicaulis*, but differs in having a densely-pubescent stem (versus slightly pubescent), ovate or broadly elliptic stipules (versus lanceolate, elliptic or subtriangular), a longer axis of inflorescence, 1–3 cm long (versus 0.3–0.5 cm long), the corolla dark purple turning blackish-purple when dried (versus purplish-pink or pale pink turning pale yellow when dried), a larger standard, ca. 12 × ca. 14 mm (versus 8–9 × 8.5–9 mm), wing petals, ca. 16 × ca. 8 mm (versus 10–11 × 3–4 mm), keel petals 11–12 × 3–4 mm (versus 9–10 × 2–2.5 mm) and a hirsute fruit stalk (versus slightly puberulous).

**Figure 1.**
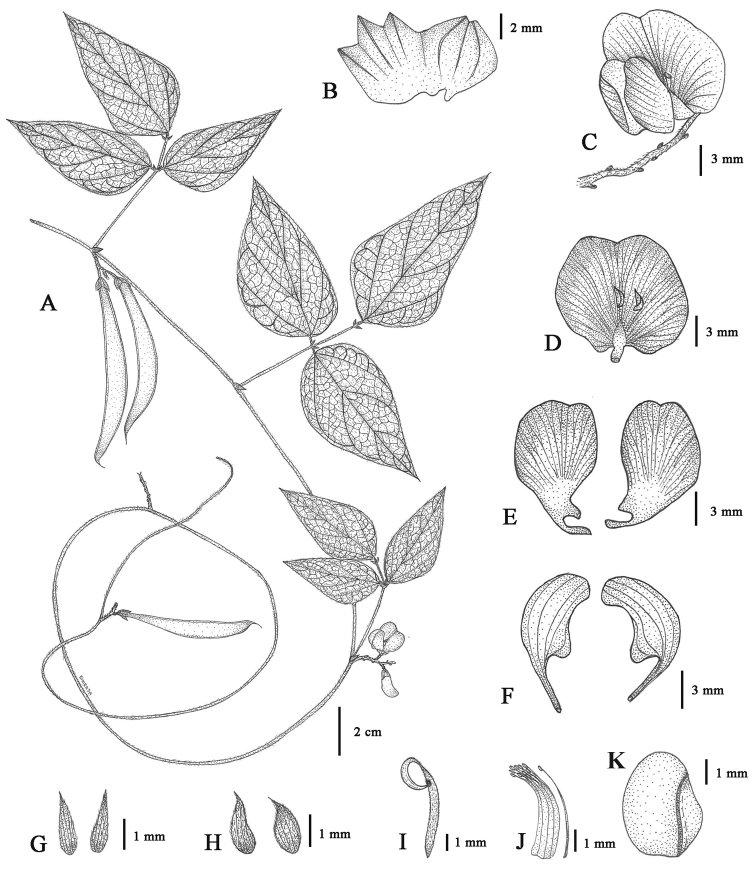
*Dolichos kongkandae* R. Meeboonya, Ngerns. & Balslev **A** stem with leaves, inflorescence and infructescences **B** calyx **C** inflorescence with a flower **D** standard petal **E** wing petals **F** keel petals **G** bracts of fascicle **H** bracteoles **I** pistil **J** stamens **K** seed. Drawn from *R. Meeboonya & P. Yodboplub 406* (BKF) by Rumrada Meeboonya.

##### Type.

Thailand. Chiang Mai, Chiang Dao, Doi Chiang Dao Wildlife Sanctuary, near the beginning of Khun Huai Mae Kok Ranger Station (Den Ya Khat) nature trail, 24 Nov 2017, *R. Meeboonya & P. Yodboplub 406* (holotype BKF!; isotypes AAU!, BK!).

**Figure 2.**
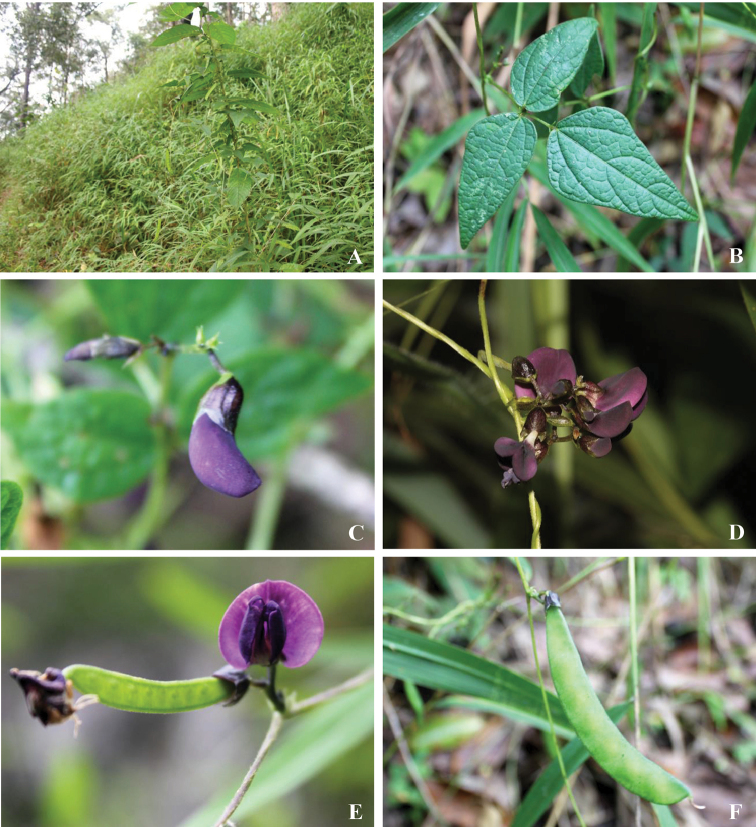
*Dolichos kongkandae* R. Meeboonya, Ngerns. & Balslev **A** habit **B** leaf **C** inflorescence with flower buds **D** inflorescence with young and mature flowers **E** inflorescence with a mature flower and a young fruit **F** infructescence with a mature fruit. Photographs from *R. Meeboonya & P. Yodboplub 406* (BKF).

##### Description.

Perennial herb, stem slender, twining, densely pubescent. ***Stipules*** persistent, ovate or broadly elliptic, 3–5 × 1.5–3 mm, with striate veins, apex acute, base subcordate or truncate, abaxial surface pubescent, adaxial surface glabrous. *Leaves* trifoliolate pinnately compound, alternate, petiole 2.5–7 cm long, sparsely pubescent; rachis 0.8–1.8 cm long, sparsely pubescent. ***Leaflets*** densely pubescent on both surfaces, with 4–6 lateral veins on each side; terminal leaflet ovate, lanceolate or broadly ovate, 3.5–8 × 1.5–4.5 cm, apex acute and apiculate, base rounded, obtuse or subcordate, margin entire; lateral leaflets slightly obliquely ovate or obliquely lanceolate, 3.5–7 × 1.2–3.5 cm, apex acute and apiculate, base rounded, obtuse or subcordate, margin entire; stipels persistent, attached to the base of petiolules of leaflets, elliptic, lanceolate or obovate, 2–2.8 × 1–1.2 mm, with striate veins, apex acute, base truncate, abaxial surface hirsute, adaxial surface glabrous; petiolule 1.5–3 mm long, densely pubescent. ***Inflorescences*** axillary, with 2–10 fascicles arranged in nodose-pseudoracemes or nodose-pseudopanicles; peduncle 2–5 mm long, sparsely hirsute; axis 1–3 cm long, hirsute; bracts of fascicle 2, persistent, lanceolate, 2–2.5 × 0.5–1 mm, with striate veins, apex acute, base obtuse, margin ciliate, abaxial surface pubescent, adaxial surface glabrous. ***Flowers*** 1 in each fascicle; pedicel 2–3 mm long, densely hirsute; bracteoles 2, attached near the apex of pedicel, ovate, 1.8–2 × 0.8–1 mm, apex acute or acuminate, base obtuse, margin ciliate, abaxial surface pubescent, adaxial surface glabrous. ***Calyx*** greenish-purple, campanulate, 5-lobed, calyx tube ca. 4.5 × ca. 5.5 mm, 2 upper lobes connate, apex slightly divided to shallow lobes, the lowest of 3 lower lobes deltoid, ca. 2 × ca. 2.5 mm, apex acute, 2 lateral lobes deltoid, ca. 1 × ca. 1.5 mm, apex acute, abaxial surface sparsely pubescent, adaxial surface glabrous. ***Corolla*** pentamerous, dark purple, turning blackish-purple when dried, with clawed petals; standard petal, suborbicular, ca. 12 × ca. 14 mm, apex emarginate, near the centre with 2 appendages on adaxial surface, 1.5–2 mm long, base with 2 small auricles, ca. 1 mm long, the claw white, 1–2 mm long; wing petals, obovate, ca. 16 × ca. 8 mm, apex emarginate, base with 1 appendage, 1.5–2 mm long, the claw white, ca. 4 mm long; keel petals oblong, 11–12 × 3–4 mm, pubescent along the inner margins, apex truncate, the claw white, ca. 4 mm long. ***Stamens*** 10, diadelphous, 9 filaments connate and a vexillary filament free, filaments white, ca. 10 mm long; anthers uniform and dorsifixed, yellow, oblong, ca. 0.5 mm long. ***Pistil*** simple, ovary superior, ca. 9 mm long; ovary light green, linear, pubescent, base shortly stipitate; style flattened; stigma capitate with long hairs. ***Fruits*** oblong, slightly falcate, 5–7.5 cm × 6–8 mm, glabrous or slightly pubescent along both margins, apical beak 3–5 mm long, base stipitate, 2–3 mm long; fruit stalk 3–5 mm long, hirsute. ***Seeds*** 5–8, young seeds light green, dry seeds brown, elliptic or oblong, compressed, ca. 4 × 2.5–3 mm.

##### Phenology.

Flowering and fruiting from August to December.

##### Distribution.

Bhutan, India, Myanmar, China, Laos, Thailand.

##### Ecology.

Open areas in montane rain forests, mixed deciduous forests, limestone ridges, 550–2150 m alt.

##### Vernacular name.

Thua doi dok muang kongkanda (ถั่วดอยดอกม่วงก่องกานดา), the name is here given by the authors. This vernacular name references legumes (*thua*), the hills or mountain regions of its origin (*doi*), purple corolla (*dok muang*), and our mentor (Dr. Kongkanda Chayamarit).

##### Conservation status.

*Dolichos kongkandae* is widely distributed in its habitats. However, these areas are disturbed by the human activities. It is therefore considered as Near Threatened (NT), following the IUCN Red List Criteria and Categories version 14 (IUCN 2019).

##### Etymology.

The specific epithet is named in honour of Dr. Kongkanda Chayamarit, the expert botanist of the Forest Herbarium and the Flora of Thailand Project. She was the former supervisor of Associate Professor Dr. Chatchai Ngernsaengsaruay in his master’s and doctoral degrees and the thesis co-advisor of Dr. Rumrada Meeboonya in her master’s and doctoral degrees. She has always encouraged and supported us.

##### Additional specimens examined.

**Bhutan.** Kauchaw, Punakha, 21 Aug 1914, *R.E. Cooper & A.K. Bulley 3279* (BM!, E!); 26 Aug 1915, *R.E. Cooper & A.K. Bulley 4627* (BM!, E!). **India.** Assam, Karong, Manipur, 26 Sept 1950, *W.N. Koelz 26277* (L!); 9 Oct 1869, *C.B. Clarke s.n.* (K!); 3 Oct 1875, *C.B. Clarke 24925* (BM!, K!). **Myanmar.** Mandalay, Maymyo plateau, 3 Oct 1908, *J.H. Lace 4270* (E!, K!). **China.** Yunnan, Aug 1912, *G. Forrest 8821* (E!); ibid., Aug 1913, *G. Forrest 11966* (E!). **Thailand.** Chiang Mai: Doi Chiang Dao, 2 Nov 1922, *A.F.G. Kerr 6514* (BK!, BM!, E, K); ibid., 14 Oct 1926, *Put 322* (AAU!, BK!, BM!, E!, K!); ibid., 9 Nov 1962, *T. Smitinand*, *M.E.D. Poore & R.G. Robbins 7742* (BKF!); ibid., 25 Sept 1971, *G. Murata*, *K. Iwatsuki & C. Phengklai T-14920* (BKF!); ibid., 25 Sept 1971, *G. Murata*, *K. Iwatsuki & C. Phengklai T-14930* (AAU!, BKF!, L!); ibid., 25 Sept 1971, *J.E. Vidal 5161* (AAU!, P!); ibid., 27 Sept 1971, *J.E. Vidal 5233* (AAU!, P!); ibid, 27 Oct 1979, *T. Shimizu*, *H. Toyokuni*, *H. Koyama*, *T. Yahara*, *T. Santisuk & C. Niyomdham T-21180* (BKF!); ibid., 4 Nov 1995, *J.F. Maxwell 95-1051* (BKF!, CMUB!, L!); ibid., Dec 2000, *T. Rotjanadirok 81* (CMUB!); ibid., 12 Nov 2011, *R.P. Clark*, *P. Wilkin*, *P. Suksathan*, *A. Trias-Blasi & Phitak 211* (K!); ibid., 12 Nov 2011, *R.P. Clark*, *P. Wilkin*, *P. Suksathan*, *A. Trias-Blasi & Phitak 230* (K!); ibid., 11 Nov 2012, *V. Chamchumroon*, *M. Callmander*, *S. Christoph*, *C. Davidson*, *J. Regalado*, *S. Sirimongkol*, *N. Ritphet & S. Lai-lung 5564* (BKF!); ibid., 7 Nov 2013, *A.N. Egan*, *R.P. Clark*, *S. Sirimongkol*, *V. Chamchumroon & R. Meeboonya 13-0806* (K!); Mae Taeng, 30 Oct 1922, *A.F.G. Kerr 6491* (BK!, BM!, C!, K!).

##### Notes.

**Figure 3.**
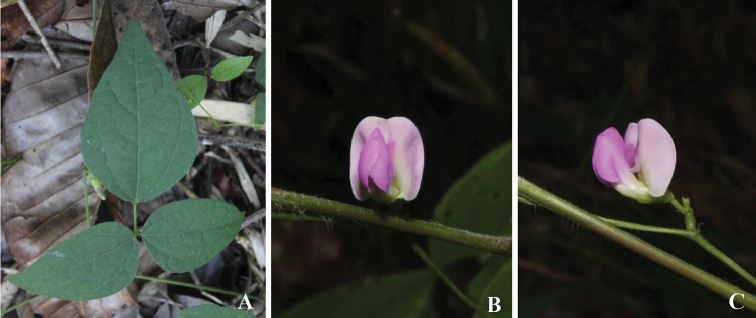
*Dolichos tenuicaulis* (Baker) Craib **A** leaf **B, C** inflorescence with a mature flower. Photographs from *R. Meeboonya*, *K. Kommongkol*, *T. Napiroon*, *P. Wessapak & P. Yodboplub 372* (BKF).

*Dolichos kongkandae* has been usually confused with *D. tenuicaulis* (Fig. 3) because of some morphological similarities. This is especially true of dried herbarium specimens.

The type specimens of *Phaseolus tenuicaulis* Baker (the basionym of *D. tenuicaulis*), *Wallich Cat. no. 5598 D* (holotype K001121419!), has a slightly pubescent stem, lanceolate, elliptic or subtriangular stipules, shorter axis of the inflorescence and the corolla turning pale yellow when dried. It also has a smaller standard, wing and keel petals and a slightly puberulous fruit stalk all of which distinguish it from *D. kongkandae* (Table 1).

**Table 1.** Morphological differences between *Dolichos kongkandae* and *D. tenuicaulis*.

Characters	*D. kongkandae*	*D. tenuicaulis*
Stem indumentum	densely pubescent	slightly pubescent
Stipules shape and width	ovate or broadly elliptic, 1.5–3 mm wide	lanceolate, elliptic or subtriangular, 1–1.5 mm wide
Leaflets indumentum	densely pubescent	slightly pubescent
Stipels shape and width	elliptic, lanceolate or obovate, 1–1.2 mm wide	elliptic, up to 0.5 mm wide
Inflorescence axis length and indumentum	1–3 cm long, hirsute	0.3–0.5 cm long, slightly puberulous
Bracts of fascicle shape and size	lanceolate, 2–2.5 × 0.5–1 mm	lanceolate or ovate-lanceolate, 1.5–1.8 × ca. 0.5 mm
Corolla colour	dark purple, turn to blackish-purple when dried	purplish-pink or pale pink, turning to yellow when dried
Pedicel indumentum	densely hirsute	puberulous
Calyx colour, shape, size and apex of the lowest of 3 lower lobes	greenish-purple, deltoid, ca. 2 × ca. 2.5 mm, apex acute	green, deltoid, ca. 1 × ca. 1.5 mm, apex obtuse and apiculate
Standard petal size	ca. 12 × ca. 14 mm	8–9 × 8.5–9 mm
Wing petals shape and size and appendage length	obovate, ca. 16 × ca. 8 mm, appendage at base 1.5–2 mm long	narrowly obovate, 10–11 × 3–4 mm, appendage at base, ca. 0.8 mm long
Keel petals size	11–12 × 3–4 mm	9–10 × 2–2.5 mm
Fruit stalk length and indumentum	3–5 mm long, hirsute	2–3 mm long, slightly puberulous

Craib (1912) published *D. tenuicaulis*, based on the specimens of *Lace 4270* and *Robertson 14*. Moreover, he cited *D. falcatus*, based on the specimens of *Housseus 45* and *Kerr 834.* We studied these specimens and found that *Housseus 45*, *Kerr 834* and *Robertson 14* are similar to *D. tenuicaulis*. However, the specimens of *Lace 4270* have a densely-pubescent stem and leaves, ovate or broadly-elliptic stipules, longer axis of the inflorescence, densely-hirsute pedicel and the corolla turns to blackish-purple when dried; it has a larger standard, wing and keel petals and a hirsute fruit stalk which is not similar to *D. tenuicaulis* and these specimens are similar to *D. kongkandae*.

Craib (1928) reported *D. tenuicaulis* as occurring in Thailand, based on the specimens *Hosseus 45*, *Kerr 834*, *Kerr 4569*, *Kerr 6491*, *Kerr 6514*, *Put 322* and *Winit 1542*. We studied these specimens and found they can be separated into two groups. The specimens *Kerr 834* from Doi Suthep, Chiang Mai Province, *Hosseus 45* and *Kerr 4569* from Nakhon Sawan Province and *Winit 1542* from Lamphun Province have slightly pubescent stem and leaves, lanceolate, elliptic or subtriangular stipules, shorter axis of inflorescence, puberulous pedicel, the corolla turning to yellow when dried, has smaller standard, wing and keel petals and slightly puberulous fruit stalk, similar to *D. tenuicaulis*, whereas *Kerr 6491*, *Kerr 6514* and *Put 322* from Chiang Mai Province are *D. kongkandae*.

We thoroughly examined the specimens of *Dolichos* from Thai and foreign herbaria. We consistently found that the herbarium specimens of *D. kongkandae* were misidentified as *D. tenuicaulis*. *Dolichos kongkandae* is distributed in Bhutan, India, Myanmar, Laos and Thailand. In Thailand, it is only found in Doi Chiang Dao and Mae Taeng, Chiang Mai Province. *Dolichos tenuicaulis* is distributed only in Myanmar and the northern, north-eastern and south-western regions of Thailand (Fig. 5).

### Lectotypification

#### Dolichos fragrans

Taxon classificationPlantaeFabalesFabaceae

Kerr, Bull. Misc. Inform. Kew 1941(1): 9. 1941.

E0C51B02-0091-5000-AE73-737C18A6A8DF

##### Type material.

**Thailand**: Doi Chiang Dao, Steep limestone peak, 14 Mar 1940, *H.B.G. Garrett 1167* (lectotype, designated here: K! [K000900658]; isolectotypes BM! [BM000839653], E! [E00275944], L! [L1952810], P! [P02775596]).

##### Notes.

**Figure 4.**
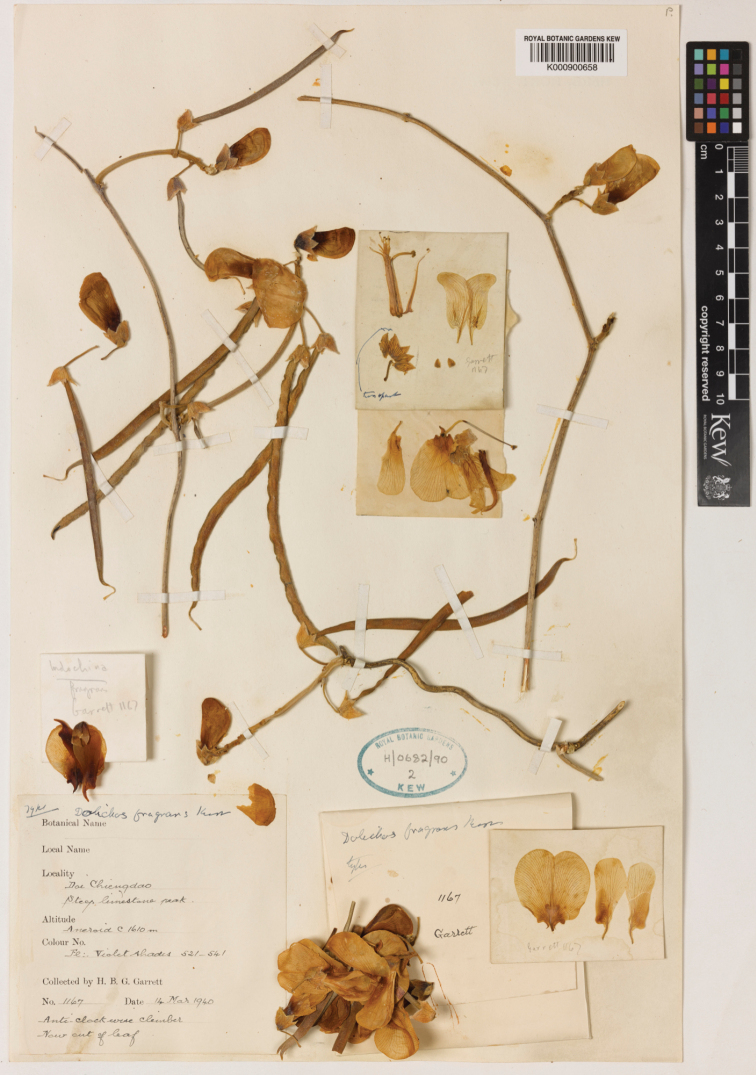
Lectotype of *Dolichos fragrans* Kerr, H.B.G. Garrett 1167 (K); see The Royal Botanic Gardens, Kew, Herbarium Catalogue (2020). http://specimens.kew.org/herbarium/ K000900658.

According to the protologue (Kerr 1941), the nomenclatural type of *D. fragrans* is *H.B.G. Garrett 1167*. Five syntypes of this gathering were deposited in BM, E, K, L and P and none of them has leaves (the leaves are often caducous when flowering). We designated the sheet K000900658 as the lectotype (Fig. 4), because it has many flowers and fruits, both of young and mature and also dissected flowers. The sheets, designated isolectotypes (BM000839653, E00275944, L1952810, P02775596), have fewer flowers and fruits than the sheet deposited at K and they have only flowers and young fruits, but no mature fruits.

The protologue also cites the specimens of *Kerr 2854*, *Put 4480* and *de Schauensee 719708*. These specimens were also collected from the limestone mountain of Doi Chiang Dao Wildlife Sanctuary and they must be regarded as paratypes, according to Article 9.7 (Turland et al. 2018).

**Figure 5.**
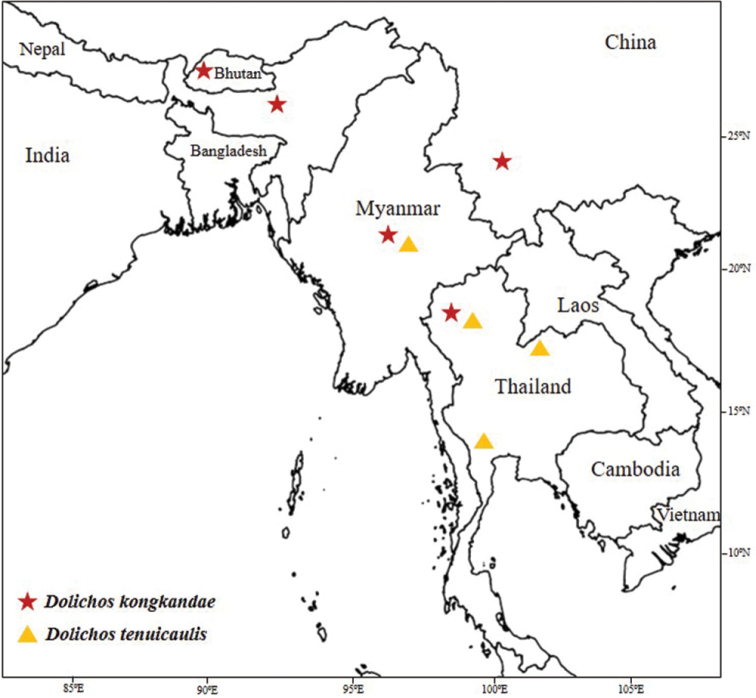
The geographical distribution of *Dolichos kongkandae* R. Meeboonya, Ngerns. & Balslev, sp. nov. and *D. tenuicaulis* (Baker) Craib.

## Supplementary Material

XML Treatment for Dolichos kongkandae

XML Treatment for Dolichos fragrans
